# Negative relationship between thermal tolerance and plasticity in tolerance emerges during experimental evolution in a widespread marine invertebrate

**DOI:** 10.1111/eva.13270

**Published:** 2021-07-13

**Authors:** Matthew C. Sasaki, Hans G. Dam

**Affiliations:** ^1^ Department of Marine Sciences University of Connecticut Groton CT USA

**Keywords:** climate change, copepod, developmental phenotypic plasticity, experimental evolution, temperature, thermal tolerance, trade‐off

## Abstract

Whether populations can adapt to predicted climate change conditions, and how rapidly, are critical questions for the management of natural systems. Experimental evolution has become an important tool to answer these questions. In order to provide useful, realistic insights into the adaptive response of populations to climate change, there needs to be careful consideration of how genetic differentiation and phenotypic plasticity interact to generate observed phenotypic changes. We exposed three populations of the widespread copepod *Acartia tonsa* (Crustacea) to chronic, sublethal temperature selection for 15 generations. We generated thermal survivorship curves at regular intervals both during and after this period of selection to track the evolution of thermal tolerance. Using reciprocal transplants between ambient and warming conditions, we also tracked changes in the strength of phenotypic plasticity in thermal tolerance. We observed significant increases in thermal tolerance in the Warming lineages, while plasticity in thermal tolerance was strongly reduced. We suggest these changes are driven by a negative relationship between thermal tolerance and plasticity in thermal tolerance. Our results indicate that adaptation to warming through an increase in thermal tolerance might not reduce vulnerability to climate change if the increase comes at the expense of tolerance plasticity. These results illustrate the importance of considering changes in both a trait of interest and the trait plasticity during experimental evolution.

## 1 INTRODUCTION

One of the fundamental challenges facing biologists is predicting how organisms will respond to a rapidly changing environment. Experimental evolution studies have become an important tool for studying the mechanisms of evolutionary change (Bennett & Lenski, 1999; Kawecki et al., 2012) and have therefore become important components in the climate change biology toolkit. Using this approach, model systems with especially short generation times have yielded fundamental insights into the role of de novo mutations in driving evolutionary change (Barrick & Lenski, 2013; Barrick et al., 2009; Herring et al., 2006). Studies have also shown that populations often have the capacity to respond to environmental change via the segregation of standing genetic variation (Orsini et al., 2012; Pespeni et al., 2013; Reusch & Boyd, 2013).

Most studies have focused on the role of genetic differentiation in producing long‐term change in phenotypes during experimental evolution. Indeed, in many cases the availability of genomic resources allows researchers to pinpoint the genetic basis for observed changes (Burke et al., 2010; Deatherage et al., 2017; Orsini et al., 2012). However, how phenotypic plasticity in the trait of interest changes over time is also crucial to consider. Phenotypic plasticity, the ability of a single genotype to produce multiple phenotypes in response to different environmental conditions, is expected to play an important role in organismal responses to climate change (Ayrinhac et al., 2004; Burggren, 2018; Chown et al., 2007; Fox et al., 2019; Kelly, 2019; Sasaki & Dam, 2019; Seebacher et al., 2014).

Several processes can result in simultaneous change in a trait and plasticity in that trait during experimental evolution. In one scenario, fixed changes in the trait of interest may stem from changes in phenotypic plasticity of that trait (e.g. genetic assimilation or ‘plasticity first evolution’; Crispo, 2008; Friedrich & Meyer, 2016; Pigliucci et al., 2006; Vigne et al., 2021; Waddington, 1959). While detecting the effects of genetic assimilation in natural populations is notoriously difficult (Levis & Pfennig, 2016, 2019), it may play an important role in adaptation to climate change (Kelly, 2019). Stable laboratory conditions may also promote changes via the loss of ancestral plasticity in a trait. The reverse is also possible, with trait changes driving changes in plasticity of the trait. Basal, or innate thermal tolerance and plasticity in thermal tolerance may be negatively related, for example, where the evolution of increased basal thermal tolerance drives a reduction in the capacity for plasticity to modify thermal tolerance (Armstrong et al., 2019; Stillman, 2003). This trade‐off is especially important to consider when using experimental evolution to make predictions about population vulnerability to climate change: an increase in thermal tolerance may not reduce vulnerability to warming if it comes at the expense of plasticity in this trait. It is well‐established that plasticity can be adaptive in variable environments (Burggren, 2018; Ghalambor et al., 2007), and warm‐adapted populations with low levels of phenotypic plasticity have been suggested as the most vulnerable to climate change (Huey et al., 2009; Sasaki & Dam, 2019; Somero, 2010; Stillman, 2003; Vale & Brito, 2015).

Despite its well‐documented effects on thermal limits, few studies have examined changes in tolerance plasticity during experimental evolution. Of those that have, warm‐adapted lineages often differ significantly from control lineages in terms of plasticity in tolerance traits (Cavicchi et al., 1995; Esperk et al., 2016; Kelly, Pankey et al., 2016; Kinzner et al., 2019; Morgan et al., 2020). While these studies generally indicate that adaptation to increased temperature reduces plasticity in thermal tolerance, additional studies are needed in diverse taxa to determine how generalizable these patterns are. Copepods are some of the most abundant metazoans in marine systems and dominate planktonic communities (Huys & Boxshall, 1991; Mauchline, 1998). As such, they play key roles in aquatic food webs and biogeochemical cycles and are especially important as a food source for larval fish (Steinberg & Landry, 2017; Turner, 2004). How copepods respond to a changing climate will affect future aquatic community dynamics (Dam, 2013). Given their ecological relevance, large natural abundances and short generation times, several species of copepods are ideal model systems for experimental evolution studies.

In this paper, we describe an experimental evolution project involving three populations of the widespread and ecologically important marine copepod, *Acartia tonsa*. Our aim is to examine how both thermal tolerance and the effects of developmental phenotypic plasticity on thermal tolerance change across generations in response to chronic nonlethal temperature differences. We utilized multiple populations to assess how general these changes may be. The duration of our experiment, the examination of changes in both tolerance and tolerance plasticity and the inclusion of multiple populations are notable among other experimental evolution studies of metazoan thermal tolerance. We show that substantial changes in both thermal tolerance and plasticity in thermal tolerance occur rapidly (within 15 generations) and that these changes may be strongly affected by a trade‐off or negative relationship between basal thermal tolerance and tolerance plasticity.

## 2 METHODS

### 2.1 Culture collection and maintenance

Copepods were collected from three sites, one in Eastern Connecticut (CT; 41.32 N, −72.00 W) and two from the Gulf Coast of Florida—St. Petersburg (SP; 27.63 N, −82.67 W) and Punta Gorda (PG; 26.94 N, −82.05 W). Mean annual temperatures varied across the three sites (CT – 13.1°C; SP – 24.02°C; and PG – 24.8°C), but there is substantial seasonal variation at all three sites as well. These sites were selected based on previous characterization of their thermal survivorship curves (Sasaki & Dam, 2019; Sasaki et al., 2019). Geographically distant sites (CT and SP) share a similar thermal survivorship curve (TSC), while PG exhibits markedly increased thermal tolerance, even compared with the geographically proximate SP site. At each site, copepods were collected in surface tows using a 250‐μm mesh plankton net with a solid cod end. All collections occurred during July or August. Within 12 h of collection, mature *A*. *tonsa* individuals were identified using a dissection microscope and sorted into 0.6 μm filtered seawater (FSW), with salinity and temperature adjusted to match collection conditions. For each population, we established six replicate cultures, each with 400 females and 100 males. Cultures were transported back to the University of Connecticut Avery Point campus by car in temperature‐controlled containers. Temperature and salinity were maintained near collection conditions. Aquarium bubblers were used to keep containers well oxygenated throughout the duration of transport. Copepods were fed a mixture of a green flagellate (*Tetraselmis* sp.) and a small diatom (*Thalassiosira weissflogii*) during transport. In the laboratory, live cultures were gradually brought to 18°C and 30 practical salinity units (psu).

### 2.2 Selection environment

A schematic of the full experimental design is shown in Figure S1. The F0 cultures were split into two groups per site. One group, designated the Control lineage, was maintained at 18°C. The second F0 group, designated the Warming lineage, was moved to 19°C. Temperature was then increased 1°C per generation for the Warming lineage through the F3 generation (reaching a final temperature: 22°C), after which cultures were maintained at constant temperature. These temperatures were selected based on previous work, which showed that all populations successfully developed and reproduced at both temperatures (Sasaki & Dam, 2019; Sasaki et al., 2019) and a general estimate of sea surface temperature increase over the next century from the IPCC RCP 8.5 scenario (IPCC, 2014). It is important to note that all three populations experience seasonal temperature cycles that at least approaches temperatures selected for laboratory culture maintenance (Sasaki & Dam, 2019). Further, because of these copepods' relatively short generation times (usually <1 month), these seasonal temperature cycles are experienced across generations, rather than within. This cyclical temperature variation may maintain adaptive genetic variation in both thermal tolerance and plasticity in thermal tolerance (Sasaki & Dam, 2020), priming populations to respond to changes in mean temperature. Rather than the effects of lethal selection on thermal tolerance, our choice of temperatures examines the capacity for standing genetic variation to respond to chronic, sublethal temperature differences.

Aquarium bubblers were used to ensure cultures were well oxygenated, and water changes occurred weekly. Generations were kept separate by collecting eggs during the weekly water changes. By collecting and pooling these eggs across several transfers, we maintained the largest possible population sizes, maintained the separation of generations and minimized selection for either fast or slow maturation times. Copepods were fed *ad libitum* several times a week with a mixture of a green flagellate (*Tetraselmis* sp.), a small diatom (*T*. *weissflogii*) and a cryptomonad (*Rhodomonas salina*). Phytoplankton were cultured semicontinuously in F/2 medium (without silica for *Tetraselmis* and *Rhodomonas*) with a 12 h:12‐h light:dark cycle at 18°C.

### 2.3 Thermal survivorship assays

We tracked the evolution of basal thermal tolerance and phenotypic plasticity in thermal tolerance across generations, following a protocol previously used to estimate these metrics in copepods (Pereira et al., 2017; Sasaki & Dam, 2019; Sasaki et al., 2019). Briefly, healthy mature females were isolated in FSW and single individuals gently transferred to a 2‐ml microfuge tube. Tubes were partially capped, allowing for gas exchange with the atmosphere but limited evaporation. Tubes were then placed into 15‐well dry heat baths (USA Scientific) and exposed to one of a range of temperatures (from 18 to 38°C). No intermediate temperature steps were included to minimize potential effects of acclimation or hardening in the acute heat stress. Only one female was placed into each tube, and each female experienced only one heat stress. The number of individuals exposed to each temperature generally ranged between 6 and 32, with fewer individuals exposed to the lowest and highest temperatures where variability in survivorship is lowest. Across the entire experiment, there were five instances when a temperature had a sample size of less than 6 when a copepod could not be found in the tube after the heat stress. After 24 h, individual survivorship was scored visually using a dissection microscope. These binary survivorship data were used to estimate thermal survivorship curves (TSCs) using a logistic regression. Thermal tolerance was estimated from these TSCs as LD_50_, the temperature inducing 50% mortality (described in greater detail in the ‘Statistical Analysis’ section below). Roughly equal numbers of individuals from the replicate cultures within each lineage were used for each heat stress. However, because of the large number of individuals required for each survivorship curve (~200 individuals per curve), we pooled the data across replicates to estimate a mean thermal tolerance rather than analysing each replicate separately. As such, the three populations are the statistical units of replication. These TSCs were generated every three generations from F3 to F15 for the Warming lineages and for the F3 and F15 generations for the Control lineages. Females were not returned to the main cultures after experiencing a thermal stress, and thus, observed changes across generations were derived from the increase in ambient temperature experienced by the Warming lineage.

### 2.4 Effects of phenotypic plasticity on TSCs

In addition to these TSCs for individuals that developed at respective ambient temperatures (Control at 18°C and Warming at 22°C), we also transplanted individuals between the two temperature conditions to quantify the effect of developmental temperature on thermal tolerance. The Warming lineage was transplanted back to the control conditions during the F3, F9 and F15 generations (F3, F9 and F12 for the PG lineage due to a clerical error). Transplants from the Control lineages to 22°C occurred during both the F3 and F15 generations. For these transplants, eggs were collected and split into two groups, which then developed at either 18 or 22°C before being exposed to the same acute heat stress assay described in the previous section. Transplants resulted in four categories of TSCs: (1) Control copepods developed in control conditions, (2) Control copepods developed in warming conditions, (3) Warming lineage copepods developed in warming conditions and (4) Warming lineage copepods developed in control conditions. By comparing the TSCs between developmental conditions within a lineage, these transplants allowed us to track how the effects of developmental phenotypic plasticity on thermal tolerance changed across the duration of the experiment, in addition to how basal thermal tolerance evolved.

### 2.5 Postselection culture maintenance

After the F15 generation, the three replicate cultures for each population x lineage combination were pooled into a single culture for long‐term maintenance at 18°C. TSCs for individuals from both lineages that developed at 18°C and 22°C were generated again for the (approximate) F40 and F80 generations, allowing us to (I) ensure that changes observed during the selection phase (F0‐F15) were due to genetic differentiation and not transgenerational plasticity and (II) to continue to observe the change in tolerance plasticity over time in a stable environment. If changes during the F3–F15 time period were the result of transgenerational plasticity, we expect to see no differences between the Control and Warming lineages in the F40 or F80 generations, after >25 generations of culturing under common conditions. All populations were included in the F40 TSCs, but the SP population was excluded from the F80 experiments after an incubator malfunction caused a sharp decrease in population size. A small number of the TSCs involved in this experiment have been previously published: for all three populations, the F3 Control lineage TSCs were published in Sasaki and Dam (2019), and the TSCs for the CT and PG F40 Controls were taken from Sasaki et al. (2019).

### 2.6 Statistical analysis

All analyses were performed using the software package R (R Core Team, 2020). Analysis of the TSCs generated throughout the experiment comprised two components. First, we compared just the TSCs from the selection phase (generations 3 through 15). TSCs were estimated as logistic mixed effect models of survival against stress temperature using the *lme4* R package. An ANOVA was used to examine the observed changes over time (survival as a function of stress temperature, generation, lineage and developmental temperature, with the interaction between lineage and developmental temperature. Population was included as a random effect). A second set of analyses focused on just the TSCs of the F40 generation. Because all cultures had been maintained at 18°C for ~25 generations at this time, this comparison tests for stable changes in basal thermal tolerance and in tolerance plasticity between treatments (Control vs. Warming). An ANOVA was again used to compare TSCs between lineages and developmental temperatures (survival as a function of stress temperature, lineage, developmental temperature, and their interaction. Population was again included as a random effect).

All TSCs generated throughout the project were further summarized by estimation of LD_50_ values (the temperature at which 50% survivorship would be expected). For transplanted generations, the difference in LD_50_ values between the two developmental temperature treatments (ΔLD_50_) represents the strength of developmental phenotypic plasticity in thermal tolerance. Standard errors for the ΔLD_50_ values were calculated as sqrt(SE_18C_^2^ + SE_22C_^2^), where SE_18C_ and SE_22C_ are the standard error estimates for LD_50_ from the 18 and 22°C developmental temperature groups within an experimental lineage, respectively. An ANOVA was used to examine (I) the change in plasticity over time (ΔLD_50_ as a function of generation, developmental temperature, and lineage, all interactions, and population as a random effect), and (II) the relationship between plasticity and thermal tolerance throughout the experiment (ΔLD_50_ as a function of LD_50_ and lineage, along with their interaction. Population was included as a random effect). We also used these thermal tolerance values in a modified reaction norm analysis, as outlined in Govaert et al. (2016), to partition the observed changes in thermal tolerance in the F40 generation into the contributions of ancestral plasticity in thermal tolerance, constitutive evolution of thermal tolerance and evolution of plasticity in thermal tolerance. To account for any background selection by the laboratory environment, we used the thermal tolerance reaction norm of the Control F40 generation rather than the ancestral reaction norm.

## 3 RESULTS

### 3.1 Selection‐phase TSCs

**FIGURE 1:**
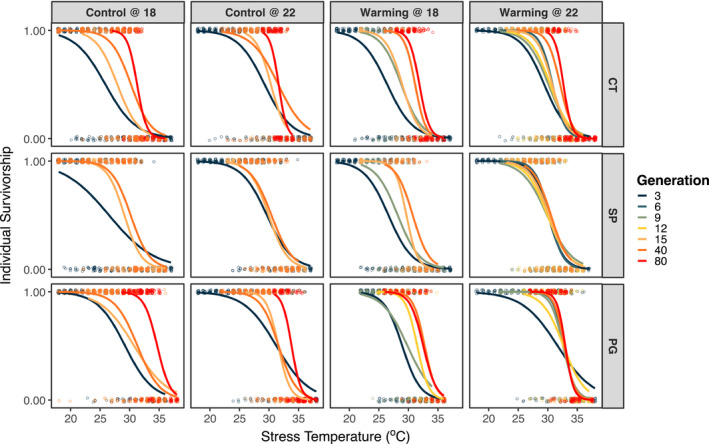
All of the thermal survivorship curves for laboratory populations of *Acartia tonsa* (Copepoda) generated during the project. Curves are separated by population, lineage, and developmental temperature. Individual survivorship measurements are shown as points, and curves are then estimated using logistic regression. The different generations are represented in different colours

Over the duration of this project, we generated 55 thermal survivorship curves (TSCs) based on 10,231 individual survivorship measurements (Figure 1). The ANOVA results for the selection phase of the experiment are shown in Table S1. All the individual factors (stress temperature, generation, developmental temperature and lineage) had significant effects. Survivorship curves generally shifted towards warmer temperatures across generations. Increased developmental temperature also resulted in shifts towards higher temperatures. However, the interaction between developmental temperature and lineage was not significant, suggesting no differences in this effect of developmental phenotypic plasticity between lineages. In general, plasticity in thermal tolerance decreased over time (Table S2; Figure S2).

### 3.2 Changes in thermal tolerance and thermal tolerance plasticity

**FIGURE 2:**
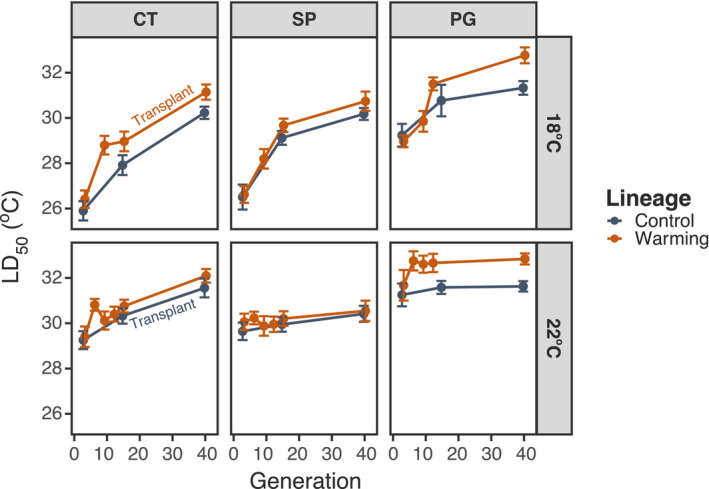
Thermal tolerance (LD_50_) shown across generations. Developmental temperatures and populations are shown separately, with experimental lineage in different colours. Warming lineage points in the 18°C developmental temperature treatment and the Control lineage points in the 22°C developmental temperature treatment constitute the transplants. This is indicated by text in the CT population column

**FIGURE 3:**
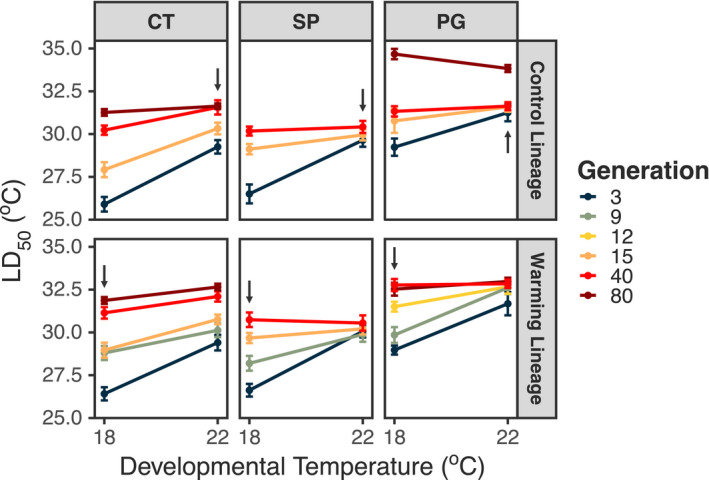
Thermal tolerance (LD_50_) reaction norms for each population x lineage combination across generations (shown in different colours). Error bars show standard errors. The slope of each norm represents the strength of phenotypic plasticity in thermal tolerance for that generation. In each facet, the arrow indicates which developmental temperature represents transplant conditions for that lineage

**FIGURE 4:**
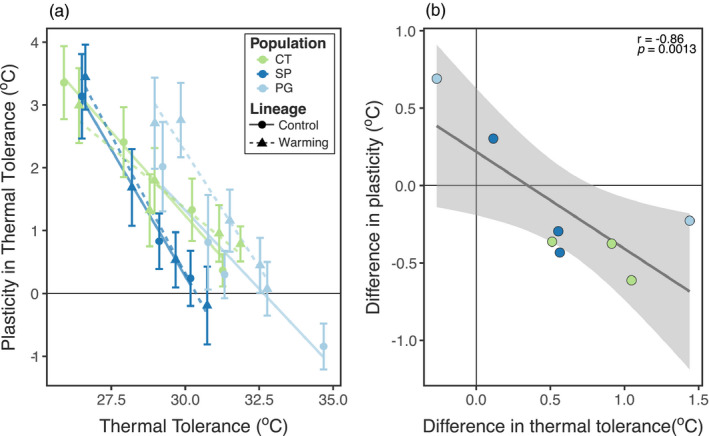
Trade‐offs between thermal tolerance and phenotypic plasticity in thermal tolerance. (a) Significant relationships are observed in the nonstandardized data. The different populations are shown in different colours, and the different lineages are plotted as different shapes. Linear regressions are shown in solid lines for the Control lineage and dashed lines for the Warming lineage. Error bars represent standard error estimates. (b) A significant correlation is also observed in the standardized comparisons between Warming and Control lineages. Between lineage comparisons were calculated as Warming lineage value − Control lineage value for both thermal tolerance and plasticity in thermal tolerance

The changes in TSCs correspond to a strong increase in thermal tolerance over time (Figure 3; Table S2). While this increase is observed in both the Control and Warming lineages (discussed further below), there was still a significant difference between the Warming and Control lineages (Figure 2; Tables S1 and S2). There was also a marked decrease in the strength of phenotypic plasticity over the course of the experiment (Figure 3; Figure S2; Table S2). These changes in tolerance plasticity are strongly correlated with the increases in thermal tolerance (Figure 4a; Table S3). There are no differences in the relationship between tolerance plasticity and tolerance between the two lineages, indicated by a nonsignificant interaction term.

Selection by the laboratory environment during long‐term culture maintenance is to be expected, and the interpretation of our results is complicated by the fact that changes occurred in both the Control and Warming lineages. To account for this, we examined the relationship between the difference in thermal tolerance between the lineages (Warming LD_50_ − Control LD_50_; Figure S3a) and the difference in the strength of developmental phenotypic plasticity (Warming ΔLD_50_ − Control ΔLD_50_; Figure S3b), shown in Figure 4b. Because fewer transplants were performed for the Control lineage, there are fewer points to compare, but we still observe a significant negative relationship between these standardized metrics (Pearson's correlation test: *r* = −0.86; *p* = 0.0013). This negative relationship indicates that larger increases in thermal tolerance in the Warming lineage relative to the Control lineage are correlated with larger decreases in the strength of phenotypic plasticity in thermal tolerance. If the loss of plasticity in thermal tolerance was driven purely by selection against plasticity by the stable laboratory conditions, we would expect no differences in tolerance plasticity between the lineages (Warming ΔLD_50_ − Control ΔLD_50_ ~ 0). While background selection by the laboratory environment likely influenced our results, observed differences between the Warming and Control lineages suggest that there was still an effect of the different temperature treatments.

### 3.3 Postselection TSCs

The ANOVA results for just the F40 generation are presented in Table S4. All individual factors (stress temperature, developmental temperature and lineage) were significant. The significant effect of lineage indicates the maintenance of the effects of the selection‐phase environmental conditions on thermal survivorship curves (i.e. maintained differences between the Warming vs. Control lineages even after many generations of culturing at the same temperature). A post hoc test (Table S5) indicates significant differences between copepods from the two lineages when cultured at the same temperature (Control @ 18 vs. Warming @ 18 and Control @ 22 vs. Warming @ 22). The interaction between developmental temperature and lineage was not significant, indicating no difference in tolerance plasticity between the lineages. The reaction norm analysis (Govaert et al., 2016) indicates that increases in Warming lineage thermal tolerance could be attributed to a positive influence of ancestral plasticity, evolved increases in thermal tolerance and the evolution of reduced plasticity in thermal tolerance (Figure S4).

## 4 DISCUSSION

Predicting if, and how rapidly, populations can adapt to new conditions is an important undertaking. We observed rapid changes in both basal thermal tolerance and the strength of phenotypic plasticity in thermal tolerance. Within 40 generations, thermal tolerance increased by 2–5°C (Figure 2), while plasticity decreased by ~66%–100% (Figure S2). The main result of this study is, however, the observed negative relationship between thermal tolerance and tolerance plasticity. This trade‐off has significant implications for predicting population responses to climate change and interpreting the results of experimental evolution studies. Changes in trait plasticity cannot be ignored during experimental evolution.

The rapid rate of change we observed is in agreement with the generally rapid responses to selection observed in this and other copepod taxa (Kelly, DeBiasse et al., 2016; Langer et al., 2019; Lee et al., 2011; Colin & Dam, 2005). Previous work with *Acartia tonsa* found evidence for local adaptation of thermal tolerance over various spatial scales (González, 1974; Sasaki & Dam, 2019; Sasaki et al., 2019) and over relatively short seasonal timescales (Sasaki & Dam, 2020). Further, this species is characterized by high levels of cryptic genetic diversity (Caudill & Bucklin, 2004; Chen & Hare, 2011; Sasaki & Dam, 2019). It might not be surprising therefore that changes were observed over such rapid timescales, possibly resulting from the sorting of pre‐existing genetic variation. We unfortunately lack the genetic data required to test this in our study.

Our results highlight that both thermal tolerance and plasticity in thermal tolerance are important to consider during experimental evolution. After accounting for the observed changes in the Control lineages, there was still a significant negative correlation between changes in thermal tolerance and changes in tolerance plasticity. Interestingly, this negative relationship has also been observed in patterns of the evolution of plasticity in thermal tolerance for copepods over large spatial scales and across the seasonal temperature cycle (Sasaki & Dam, 2019, 2020), suggesting that this trade‐off may be a widespread phenomenon in planktonic copepods. However, it should be noted that there are several other potential mechanisms besides a true mechanistic trade‐off that might result in a negative relationship between tolerance and tolerance plasticity (van Heerwaarden & Kellermann, 2020). Regardless of the mechanism behind the observed relationship, the implication is the same: adaptation to warming by increasing thermal tolerance may incur a reduction in plasticity, and thus may not reduce population vulnerability to climate change.

While the trade‐off between the two is usually framed as the evolution of increased thermal tolerance at the expense of plasticity (Stillman, 2003), if these are linked by a true mechanistic trade‐off, the evolution of increased thermal tolerance may be prevented by positive selection for plasticity in thermal tolerance. In this case, the increases in thermal tolerance we observed in a laboratory environment may be reduced in natural populations which experience a variable environment, thus maintaining selection for plasticity. In increasingly variable environments (Meehl, 2004; Stott, 2016), the ability to rapidly respond via phenotypic plasticity is a valuable attribute (Richter et al., 2011; Seebacher et al., 2014), the loss of which may have negative consequences for populations. Following this reasoning, an important alternative explanation to consider for the changes we observed is that the stable conditions employed in our study relaxed selection to maintain, or selected against, tolerance plasticity. This would then drive a correlated increase in thermal tolerance, independent of the difference in temperature experienced by the Control and Warming lineages. Adaptation to the laboratory environment has already been recognized as an important process to consider when interpreting the results of experimental evolution (Simões et al., 2008). A negative relationship between tolerance and tolerance plasticity further reinforces that selection by the laboratory environment is important to account for, as its effects on tolerance plasticity may result in misleading inferences about the evolution of thermal limits. We urge caution in the interpretation of results of experimental studies employing stable environmental conditions.

The extrapolation of results from experiments to predictions of vulnerability in various climate change scenarios needs to consider this potential trade‐off. Plasticity is likely to have its own effects on fitness under climate change scenarios (Burggren, 2018). Integrating plasticity and adaptation into models of organismal response to climate change are therefore a critical undertaking for predicting biotic responses to climate change (Donelson et al., 2019; Garzón et al., 2019; Sgrò et al., 2015; Valladares et al., 2014). This trade‐off is particularly important in the context of management and conservation aquaculture (Froehlich et al., 2017; Lorenzen et al., 2013). Any effort to supplement natural populations or generate strains with improved environmental tolerances (Carlsson et al., 2008; Fernández et al., 2014; Nguyen, 2016; Norrie et al., 2020) should consider that selection may affect both the phenotypic trait of interest and plasticity in that trait.

Our findings highlight the need for experimental evolution studies to consider that multiple mechanisms contribute to adaptive phenotypic change. Several other studies have examined changes in thermal limits in populations exposed to multiple generations of chronic thermal selection (Condon et al., 2015; Esperk et al., 2016; Geerts et al., 2015; Gilchrist et al., 1997; Kellermann et al., 2015; Kinzner et al., 2019; Manenti et al., 2015; Tobler et al., 2015). It is interesting that acute selection for increased thermal tolerance, as opposed to the chronic sublethal selection we used in this study, resulted in similar patterns in the evolution of thermal tolerance and plasticity in thermal tolerance (Morgan et al., 2020). Only a few of these studies included treatments with variable temperatures (Condon et al., 2015; Geerts et al., 2015; Kellermann et al., 2015; Manenti et al., 2015), and few studies explicitly track changes in phenotypic plasticity (Cavicchi et al., 1995; Esperk et al., 2016; Kinzner et al., 2019; Leonard & Lancaster, 2020; Manenti et al., 2015; Morgan et al., 2020). By including changes in the strength of phenotypic plasticity in the scope of experimental evolution studies, we can better understand the evolutionary processes driving phenotypic changes and better leverage these studies for predictions about population responses to climate change.

## CONFLICT OF INTEREST

The authors declare no conflicts of interest.

## Supporting information

Supplementary Material

## Data Availability

The data and R code associated with this study are openly available in a Dryad Data Repository at https://doi.org/10.5061/dryad.15dv41nxr.
