# Supplementary material for: Negative relationship between thermal tolerance and plasticity in tolerance emerges during experimental evolution in a widespread marine invertebrate
Source: Evol Appl. 2021 Jul 13;14(8):2114–23. doi: 10.1111/eva.13270 (PMC8372069; doi:10.1111/eva.13270)
Supplement: Supplementary file 1 — Supplementary Material [file EVA-14-2114-s001.docx]

Supporting Information

Tables

Supp. Table 1 - ANOVA results for selection phase thermal survivorship curves. Bolded factors are significant (p < 0.05).

|  | **Chisq** | **Df** | **Pr(>Chisq)** |
| --- | --- | --- | --- |
| **Stress Temperature** | 1820.4178 | 1 | < 2.2e-16 |
| **Generation** | 66.0243 | 1 | 4.45E-16 |
| **Dev. Temp.** | 224.2749 | 1 | < 2.2e-16 |
| **Lineage** | 10.9325 | 1 | 0.0009449 |
| Dev. Temp. x Lineage | 0.4072 | 1 | 0.5233838 |

Supp. Table 2 - ANOVA results examining the change in LD_50_ over time. Generation, developmental temperature, and lineage were included as factors, along with all interactions. Bolded factors are significant (p < 0.05).

|  | **Chisq** | **Df** | **Pr(>Chisq)** |
| --- | --- | --- | --- |
| **Generation** | 74.2452 | 1 | < 2.2e-16 |
| **Dev. Temp.** | 171.0956 | 1 | < 2.2e-16 |
| **Lineage** | 11.8386 | 1 | 0.0005801 |
| **Generation x Dev. Temp.** | 22.2934 | 1 | 2.34E-06 |
| Generation x Lineage | 2.3837 | 1 | 0.1226037 |
| Dev. Temp. x Lineage | 0.0903 | 1 | 0.763849 |
| Generation x Dev. Temp. x Lineage | 0.4843 | 1 | 0.4864714 |

Supp. Table 3 - ANOVA results for a regression between ΔLD_50_ and thermal tolerance (measured as LD_50_), lineage, and their interaction. Bolded factors are significant (p < 0.05).

|  | **Chisq** | **Df** | **Pr(>Chisq)** |
| --- | --- | --- | --- |
| **LD_50_** | 140.6973 | 1 | <2e-16 |
| Lineage | 2.3109 | 1 | 0.1285 |
| LD_50_ x Lineage | 0.2462 | 1 | 0.6198 |

Supp. Table 4 - ANOVA results for the logistic regressions from the F40 generation data. Bolded factors are significant (p < 0.05).

|  | Chisq | Df | Pr(>Chisq) |
| --- | --- | --- | --- |
| **Stress Temperature** | 586.8096 | 1 | < 2.2e-16 |
| **Dev. Temp.** | 6.5301 | 1 | 0.01061 |
| **Lineage** | 21.8404 | 1 | 2.96E-06 |
| Dev. Temp. x Lineage | 0.1525 | 1 | 0.69617 |

Supp. Table 5 – Results of a posthoc test comparing the TSCs from the F40 generation, estimated using the Tukey method with the R package emmeans.

| **Contrast** | **Estimate** | **SE** | **P-value** |
| --- | --- | --- | --- |
| control @ 18 - warming @ 18 | -0.677 | 0.197 | 0.0033 |
| control @ 18 - control @ 22 | -0.34 | 0.144 | 0.0834 |
| control @ 18 - warming @ 22 | -0.914 | 0.175 | <.0001 |
| warming @ 18 - control @ 22 | 0.337 | 0.201 | 0.3365 |
| warming @ 18 - warming @ 22 | -0.237 | 0.223 | 0.7111 |
| control @ 22 - warming @ 22 | -0.574 | 0.178 | 0.0068 |

**Figures:**

**Supp. Figure 1** - Schematic of the experimental design. Copepods were collected from the field and sorted into six replicate cultures of 500 individuals. Three cultures (denoted as Control) were maintained at 18^o^C. The remaining cultures (denoted as Warming) were maintained at 19^o^C, and then progressively warmed (1^o^C per generation) until 22^o^C was reached during the F3. Each culture symbol below indicates a thermal tolerance measurement. Labels indicate the lineage, with color indicating the developmental temperature (blue = 18^o^C, pink = 22^o^C). Transplants to opposing conditions are labeled as such. A “control transplant”, for example, indicates copepods from the Control lineage developed at 22^o^C. Thermal tolerance measurements were made every three generations for the Warming lineage and for the F3 and F15 generation for the Control lineage. Transplants occurred less frequently. After 15 generations, both Control and Warming lineages were maintained at 18^o^C. Thermal tolerance was re-assessed at the ~F40 and F80 generations for copepods that developed in both temperature conditions.

**Supp. Figure 2** - The strength of phenotypic plasticity in thermal tolerance across generations, represented as ΔLD_50_ (the difference in LD50 between copepods that developed at 18^o^C and 22^o^C). The different populations are shown in different colors, while the different experimental lineages are shown with different shapes. Regression lines are shown for the Control lineage as solid lines, and for the Warming lineage as dashed lines.

**Supp. Figure 3** - The differences between warming and control lineages for thermal tolerance (A) and the strength of phenotypic plasticity in thermal tolerance (B). The three populations are shown in different colors.

**Supp. Figure 4** – Contributions of ancestral plasticity, constitutive evolution of thermal tolerance, and evolution of plasticity in thermal tolerance, estimated using a reaction norm framework as outlined in Govaert et al. 2016 (“Eco-evolutionary partitioning metrics: assessing the importance of ecological and evolutionary contributions to population and community change”, Ecology Letters, 19:839-853). We have modified the approach to use the control lineage reaction norm rather than the ancestral norm to account for any changes driven by long-term culturing in the laboratory environment. Observed trait change for each population (warming lineage at 22^o^C – control lineage at 18^o^C) is partitioned into the contributions of the different components, shown as different color segments.
